# Severe *Plasmodium vivax* malaria among sudanese children at New Halfa Hospital, Eastern Sudan

**DOI:** 10.1186/1756-3305-5-154

**Published:** 2012-07-30

**Authors:** Hyder Mahgoub, Gasim I Gasim, Imad R Musa, Ishag Adam

**Affiliations:** 1 New Halfa Teaching Hospital, New Halfa, Sudan; 2 Faculty of Medicine, Qassim, University, Qassim, Qassim, Kingdom of Saudi Arabia; 3 Buraidah Central Hospital, Buraidah, Kingdom of Saudi Arabia; 4 Faculty of Medicine, University of Khartoum, Sudan, P.O. Box 102, Khartoum, Sudan

## Abstract

**Background:**

There are few published reports on severe *Plasmodium vivax* malaria in Africa.

**Methods:**

Clinical pattern/manifestations of severe *P. vivax* were described in children admitted at New Halfa Hospital in Sudan between September 2009-December 2011.

**Results:**

Eighteen children were admitted at the hospital during the study period with different manifestations of severe *P. vivax* malaria namely: severe anaemia (6, 33.3%), jaundice (5, 27.8%), thrombocytopenia (4, 22.2%), hypotension (3, 16.7%), cerebral malaria (2, 11.1%), epistaxis (2, 11.1%), renal impairment (1, 5.5%), hypogylcaemia and more than one manifestation (5, 27.8%)*.*

By day 2, all patients were asymptomatic, a parasitaemic and had started oral quinine and primaquine. There was no death among these patients

**Conclusion:**

Severe *P. vivax* malaria is an existing entity in eastern Sudan. Further studies are required to understand emergence of severe *P. vivax* malaria.

## Background

Malaria remains one of the most important parasitic infections in the world, with almost 225 million cases of infection and 0.78 million deaths in 2009, mainly in Africa, Asia and South America [1]. *Plasmodium vivax* is the second most common cause of malaria in the world after *Plasmodium falciparum*, moreover, *P. vivax* has a wider geographical distribution, where more people are at risk of infection (2.85 billion) [2], and it is more difficult to control because of the hypnozoite forms of the parasite [3,4]. Recent reports on *P. vivax* infections suggest that this parasite may be evolving and adapting to new epidemiological contexts, becoming not only more virulent but also more frequent in countries where the incidence has traditionally been low [3,5,6]. Furthermore, it has been shown that *P. vivax* is able to infect even Duffy-negative African patients [7].

This previous old paradigm of *P. vivax* as “benign tertian malaria” has been challenged recently by recent reports and documentation of severe *P. vivax* disease, and even deaths due to *P vivax* mono-infections [8-12]. Interestingly, in one of these studies, *P. vivax* malaria was confirmed by polymerase chain reaction (PCR) [12]. The vast majority of these reports on severe *P. vivax* malaria are from south East Asia and India, there are few published data on severe *P. vivax* from Africa [13]. The current study was conducted at New Halfa hospital in the eastern Sudan during the period of September 2009*-*December 2011 to investigate manifestations of severe *P. vivax* among children so as to add to the previous studies on severe malaria and its treatment in Sudan [14-17]. Such data is of paramount importance for the care givers, health planners and for controlling the disease e.g. by using an effective drug and eradicating this species. *P. falciparum* (95%) was the main species in the area and *P. vivax* was rare and constituted only 3% of the species in the area [18].

## Methods

Children with symptoms and signs of malaria including: fever, chills, malaise, headache, vomiting or other systemic complaints were included in this study after informed consent was obtained from the parents/adolescent themselves. Then the details of the medical history were gathered for each patient (age, sex, axillary temperature, weight, fever history) using questionnaires. Children with one or more of the manifestations of severe malaria according to the World Health Organization [19] criteria, which include cerebral malaria (unarousable coma), convulsion (more than two per 24 hours), hypotension (systolic blood pressure < 80 mmHg with cold extremities), severe anaemia (haemoglobin < 5 gm/dl), jaundice (detected clinically or bilirubin > 3 mg/dl), hypoglycaemia (blood glucose < 40 mg/dl), hyperparasitaemia (parasite count > 100,000 asexual forms/μl) and severe thrombocytopenia (<50,000/μL) were managed according to the WHO guidelines, and the rest were considered as uncomplicated cases [19].

Thin and Thick blood films were prepared and stained with 10% Giemsa, and 100 oil immersion fields were examined. The parasite density was evaluated by counting the number of asexual parasites for every 200 leukocytes, assuming a leukocyte count of 8000 leukocytes/μl. All slides were double-checked in a blinded manner and only considered negative if no parasites were detected in 100 oil immersion fields. Blood glucose was measured at baseline before quinine infusion, two hours after quinine infusion and if there was clinical suspicion of hypoglycaemia using the bedside device Accu-Chek™ Multiclix (Roche diagnostics, Mannheim Germany). The Accu-Chek™ machine was calibrated weekly and every time a new box of test strips was opened. Blood indices were performed by hematology analyzer.

Resuscitation and supportive management were given according to the WHO guidelines [19]; i.e. quinine infusion at 10 mg/kg three times a day over 2–3 hours changed to oral quinine tablets when the patient could tolerate them, correction of hypoglycaemia with 10% glucose, termination of convulsions with intravenous diazepam if this persisted for more than three minutes. Paracetamol was given every 6 hours until defervescence. Those with severe anaemia (haemoglobin < 5 g/dl) and respiratory distress were transfused with blood screened for hepatitis and HIV. Vital signs were measured every 15 minutes for the first hour, then every 2 hours until 24 hours, and thereafter every 6 hours until the discharge from the hospital. Baseline investigations were performed for every patient on admission and repeated when clinically indicated. These included levels of haemoglobin, serum urea, serum creatinine, and serum bilirubin as well as the white blood cell count. Patients were discharged home on oral quinine tablets (10 mg/kg every eight hours till day 7) and primaquine tablets for 15 days.

## Ethics

The study received ethical clearance from the Research Board of the Faculty of Medicine, University of Khartoum, Sudan.

## Results

Among 298 children diagnosed with malaria at the paediatric ward, 79 (18.0%) fulfilled one or more of the WHO criteria for severe malaria [19]. Out of these 79 children with severe malaria, 61 (77.2%) were severe *P. falciparum* malaria and 18 (22.8%) [10, 55.6% were male] had various manifestations of severe *P. vivax* malaria namely: severe anaemia (6, 33.3%), jaundice (5, 27.8%), thrombocytopenia (4, 22.2%), hypotension (3, 16.7%), repeated convulsions (3, 16.7%), cerebral malaria (2, 11.1%), epistaxis (2, 11.1%), renal impairment (1, 5.5%), hypogylcaemia and more than one manifestation (5, 27.8%). There was no death among these patients*.*

Three patients with severe anaemia received blood transfusion. Out of the six patients with severe anaemia; two patients had jaundice, one patient had hypotension and thrombocytopenia. Two patients had an enlarged spleen. All children were febrile. Different symptoms such as nausea (14, 77.8%), vomiting (9, 50%), aches (8, 44.4%), sweating (7; 39.0%), headache (7; 39.0%), and diarrhoea (3; 16.7%) were observed among these children. All of these cases were *P. vivax* mono-infections.

The admission characteristics of these children are shown in Table 1. None of the patients developed hypoglycaemia during quinine treatment. By day 2, comatose patients were fully conscious and all patients were symptom free and a parasitaemic. All patients started oral quinine and primaquine tablets within two days.

**Table 1:** **The admission variables of the Sudanese children with severe** ***P. vivax*** **malaria (*n*** **= 18) at New Halfa Hospital, Eastern Sudan**

**Variable**	**Mean (standard deviation)**
Age, years	4.9 (2.4)
Duration of illness, days	2.8(1.6)
Weight, Kg	13.2(4.4)
Height, cm	96.1(19.7)
Temperature, C	38.3(1.1)
Haemoglobin, g/dl	7.8(2.6)
White blood cells	5.6(2.1)
Parasitaemia, geometric mean parasite/μ/l	14280
Blood glucose, mg/dl	102(38.1)
Urea, mg/dl	40.6(16.8)
Creatinine, mg/dl	0.9(0.3)
Bilirubin	1.9(1.4)

## Discussion

The current study documented the severe manifestations of *P. vivax* malaria (anaemia, jaundice, hypotension, thrombocytopenia, repeated convulsions, cerebral malaria, epistaxis, renal impairment and hypogylcaemia) in an area characterized by *P. falciparum* malaria [18]. We have previously observed the same manifestations of severity that were due to *P. falciparum* malaria in the same pediatric ward as well as in the different regions of Sudan [14-17]. Only 3% of malaria cases (99/1539 blood films from 190 individuals) in the same setting were *P. vivax* while 95% were *P. falciparum* malaria giving the rate of *P. vivax/P. falciparum* malaria of 0.03 [18]. Perhaps there is an increase in the *P. vivax* malaria due to influx of Ethiopian and Eritrean populations through the border following the peace and construction of Asphalt roads between these countries and Sudan. Recent reports showed that most of the malaria infections in Ethiopia were *P. vivax* infections and even with reported treatment failure [20-22]. Our data therefore confirmed the findings of some authors, who also believe that *P. vivax* in not a rare disease in Africa and might use receptors other than Duffy to invade erythrocytes [20-24].

The manifestations of severe *P. vivax* malaria in this setting are similar to the severe manifestations of *P. vivax* malaria that has been reported from India [10,12,25-28], Brazil [29], Papua New Guinea [8,30] and Indonesia [31] both in adults and children.

Generally, according to the WHO guidelines, cerebral malaria, severe anaemia, severe thrombocytopenia and pancytopenia, jaundice, splenic rupture, acute renal failure and acute respiratory distress syndrome are the expected manifestations of severe *P. vivax* malaria [32]. Severe anaemia and acute pulmonary oedema are not uncommon. The underlying mechanisms of severe manifestations are not fully understood. It worth mentioning that there are no specific manifestations/treatment of severe *P. vivax* malaria, but according the WHO guidelines, prompt and effective treatment and case management should be the same as for severe and complicated *P. falciparum* malaria [32].

In the current study patients were asymptomatic within two days following the treatment with quinine. According to the WHO new guidelines intravenous artesunate is superior to quinine in the treatment of severe malaria [32]. Compared to quinine, intravenous artesunate has a lower risk of hypoglycaemia, significantly reduces the risk of death from severe malaria, and does not require rate controlled infusion/cardiac monitoring [33]. Yet, intravenous artesunate is not registered and available in Sudan. Primaquine is currently the only drug available for radical cure of *P. vivax* and can be safely used in children 1 to 10 years of age [34].

## Conclusion

Severe *P. vivax* malaria is an existing entity in eastern Sudan. Further studies involving clinical and molecular research are required to understand emergence of severe *P. vivax* malaria.

## Competing interests

The authors declare that they have no competing interests.

## Authors’ contributions

HM and IA designed the study. GIG and ER carried out the study and participated in the procedures. All the authors read and approved the final version.
